# Attribution of Selfhood Based on Simple Behavioral Cues: Toward a Pars‐Pro‐Toto Account

**DOI:** 10.1111/cogs.70188

**Published:** 2026-03-10

**Authors:** Jan Pohl, Kristina Nikolovska, Dennis Küster, Francesco Maurelli, Arvid Kappas, Bernhard Hommel

**Affiliations:** ^1^ Faculty of Psychology Dresden University of Technology; ^2^ Adaptive Systems Group Humbold University of Berlin; ^3^ School of Computer Science and Engineering Constructor University; ^4^ Faculty of Mathematics and Computer Science University of Bremen; ^5^ Department of Psychology Shandong Normal University

**Keywords:** Self, Mind, Attribution, Perception, Overgeneralization, Human–robot interaction, Nonhumanoid robot

## Abstract

While the necessity of a concept of “self” for understanding human behavior remains subject to debate, it evidently has significance in everyday life: Lay individuals ascribe selves to humans but also to animals and technical systems, shaping their interactions accordingly. The literature suggests that there are distal behavioral cues eliciting this perception of selfhood and they may be as minimal as simple movement observed as causal. We aimed to identify which types of behavioral cues increase selfhood‐attribution to other agents such as robots. Specifically, we compared behavior of nonhumanoid robots suggesting either the presence or absence of behavioral cues for one of the characteristics of causality, equifinality, behavioral efficiency, learning sensitivity, and context sensitivity. Results showed a consistent pattern of increased selfhood‐attribution toward robots exhibiting any one of the examined minimal characteristics. Furthermore, most perceived sentient characteristics of the robot were triggered by any single characteristic's cue. These results reflect a Halo effect like pattern: Even a single perceived cue of selfhood‐related characteristics may be sufficient to trigger a change in overall selfhood‐attribution to robots. We suggest two versions of a Brunswikian model of selfhood‐judgment, wherein selfhood is attributed based on the perception of (probably loosely defined) self‐related characteristics. We propose that not all characteristics are directly perceived by their corresponding behavioral cues; rather, that the characteristics interact with each other and/or distal cues trigger the perception of more than one characteristic. We term this a Pars‐Pro‐Toto account as people go way beyond the perceived information when attributing selfhood.

## 1 Introduction

The concept of the self refers to the idea that humans possess something that underlies the continuity of the individual and the perception of both the individual him‐ or herself and the individual's social environment. Scientific analyses commonly presuppose that something like “a self” exists and that its internal workings, its neural correlates, and its interactions with other psychological processes require investigation. Some theorists are more skeptical, however. Hume (1739) has argued that “the self” merely reflects the bundle of perceptual impressions referring to a given person him‐ or herself, that is, the totality of stimuli that a perceiver or actor actively generates and perceives—an idea that philosophers have coined the minimal self (Gallagher, 2000). Buddha was less skeptical about the actual existence of something that may be called the self, but he strongly recommended getting rid of it by means of meditation (Harvey, 2012). But today, “the self” is often regarded as a particularly colorful bundle of concepts (Hafner et al., 2022; Strawson, 2017), ranging from the minimal self to a person's narrative sense of self (Schechtman, 2011), while there are also different notions for each of these views. The self as a narrative self, for example, can be understood in a hermeneutical view. As Schechtman summarizes, proponents view the self as an agent in time, and agency cannot exist without a narrative sorting behavior in time and context. Conversely, Dennett (1992) compares the narrative self with the concept of a “center of gravity” in physics, which is merely an idea that guides our understanding of the world, yet does not have a tangible existence. In this sense, the narrative self is only a tool used by the human brain to maintain a record of its past experiences. A comparable idea can be found in Damasio (1998) notion of a biological self, which differentiates between core and extended consciousness. The former is described as awareness and (language‐independent) thought bringing the capability to act. Extended consciousness, on the other hand, is seen by Damasio as contextualizing these thoughts in time within an “autobiographical self.” While this concept is similar to the narrative self, in this view, the origin of the feeling of selfhood is not found in a sense of agency but in the ability to experience.

The list of the different notions of the self could continue almost indefinitely: Entire research traditions not limited to philosophy but also, for instance, in social and differential psychology (e.g., Baumeister, 1987; Bennett & Hacker, 2022; Kollakowski, Mammen, & Paulus, 2023), have offered definitions of different types and components of the self. Still, whether something like a coherent self‐representation actually exists or is a necessary prerequisite of human existence and intentional action, as commonly assumed by self‐theorists (e.g., Brown, 1998), must be considered an open question. For example, Kollakowski and colleagues (2023) compared various theories of selfhood in infancy and arrived at the conclusion that the idea of a self may not necessarily be a useful one. Indeed, one can argue, that when a concept has received so much attention since the beginning of philosophy and when it has been debated throughout the history of the cognitive sciences with no consensus emerging, it should rightfully be questioned—there simply might be no self. Metzinger (2004), for example, does not assume that a self exists but that human and nonhuman organisms represent themselves with a model that only turns into a selfhood‐model because it is partly conscious and not perceived as a representation—creating the subjective experience of existence and identification. Along the same lines, Braitenberg (1986) has cautioned that human cognition and behavior might emerge from the interaction of comparatively simple cognitive mechanisms rather than the workings of some overarching system like a “self.” Indeed, recent research supports this idea that cognition is not limited to the brain and neural level, rather, that it is the product of an interaction of multiple cellular and bodily systems (e.g., Ciaunica, Shmeleva, & Levin, 2023; Ruetten et al., 2025). Hence, this concept of the self might be considered a mere social construction rather than referring to some internal system or mechanism. Bennett and Hacker (2022) argue in the tradition of Wittgenstein (1953) that the use of the word “self” leads to the assumption of something measurable that does not necessarily exist and they suggest a more feasible approach is to focus on the use of this concept. This may bring to mind topics like Theory of Mind (e.g., Frith & Frith, 2005) or the intentional stance (Dennett, 1989), but they go beyond the scope of the present paper. While these concepts describe use cases for applying the concept of a self to others. The intentional stance, for example, is about how we attribute mental states to other agents in order to predict their behavior (see, Perez‐Osorio & Wykowska, 2020). In contrast, the aim of the present study is not to make any assumptions about the concept of selfhood itself and what purpose it serves—rather it tries to characterize how and when we start attributing it to other agents.

### 1.1 Background

Irrespective of whether people see themselves as having a self and whether that self‐representation does or does not play an important functional role in human cognition and decision‐making, there can be little doubt that both scientists and laypeople make use of the concept of a self in describing *other* agents. Hence, they perceive various selfhood‐related mental capacities, like body ownership, agency, experience or consciousness, personality, intentionality, and intelligence among many more (see, e.g., Gallagher, 2013, for an overview), in some agents but not in others; as, for instance, revealed by research on anthropomorphism (Epley, Waytz, & Cacioppo, 2007). This research shows that people show a surprising ease with which they imbue nonhuman agents with such human‐like aspects of selfhood, ranging all the way from God to geometric shapes and computer‐animated blobs (Epley et al., 2007). For instance, humans effortlessly attribute higher cognitive functions (Eddy, Gallup, & Povinelli, 1993) and emotions (Morris, Knight, & Lesley, 2012; Wilkins, McCrae, & McBride, 2015) to animals, or ascribe personality to dogs (Fratkin, Sinn, Patall, & Gosling, 2013; Gosling, Kwan, & John, 2003). Moreover, the Media Equation (Reeves & Nass, 1996) states that people process experiences with technological agents the same way as they would process experiences with humans. For instance, Nass, Moon, and Carney (1999) showed that people apply the principles of politeness observed between humans to computers as well. When evaluating a specific computer, responses were kinder and more homogeneous (both verbal and in text) when the responses were given to the exact same computer as compared to another computer of the same model or with different response formats like pen‐and‐paper. In other words, when evaluating a computer face‐to‐face, participants would adhere to rules of politeness, but when giving the evaluation to a third party, their responses were more honest and straightforward.

When even computers elicit anthropomorphism, it seems not surprising that the same is true for robots. It has been shown, for example, that people ascribe emotions to robots both explicitly and implicitly (Spatola & Wudarczyk, 2021), furthermore, that people interact with robots according to their portrayal of specific emotions (Lakatos et al., 2014). Imaging studies have also shown that even nonhumanoid robots, when smiling, can elicit similar early brain potentials (P1, N170) to that of photos of a smiling person (Dubal, Foucher, Jouvent, & Nadel, 2011). Thellman, Graaf, and Ziemke (2022) found by reviewing the literature that there is an increase in ascribing mental states from computer to robot and that the attribution is modulated, among other factors, by behavior and appearance of the robot. Indeed, Martini, Gonzalez, and Wiese (2016) have shown that for humanoid robots the anthropomorphic visual features are sufficient to trigger an increase in mind attribution as measured by concepts like agency, emotions, and cognitive skills—in other words, features related to selfhood. Similarly, Gray, Gray, and Wegner (2007) identified the two factors agency and experience in mind perception. Experience included capacities such as consciousness, personality, or basic emotions. An alternative framework for mind perception by Weisman, Dweck, and Markman (2017) suggests that the attribution of mental states is driven by the three factors “body,” “heart,” and “mind.” The factor body refers to physiological sensations, such as hunger and pain, but also to consciousness and intentions. Heart, in this framework, refers to basic and social emotions as well as moral judgments. The last factor, mind, corresponds to perceptual abilities and information processing. Overall, this illustrates that there are many dimensions in the literature on anthropomorphism and mind perception closely linked to concepts relevant in the definition of selfhood (as, e.g., described in Gallagher, 2013).

Anthropomorphism is such a strong tendency in humans, that even abstract agents or doors will be interpreted through the human scheme. Ju and Takayama (2009) found, that manipulating the speed and trajectory of a door was sufficient to trigger an interpretation of different “door gestures,” as well as the attribution of cognition and intent to the door. A more famous example is the classical study by Heider and Simmel (1944). In their study, participants were asked to freely describe what was happening in a short, animated film showing three moving shapes, a large and a small triangle and a circle, as well as a rectangle that was static except for a section that could be suggesting a door. What Heider and Simmel observed was that all but one of the participants described the movement of the shapes as the actions of animate beings, and for the majority even as the actions of people. It appears that the shapes' movements alone led to perceptions of intentions and motivations, indicating that there are recognizable behavioral cues for selfhood‐attribution.

At this point, the reader may inquire as to whether the phenomenon in question can truly be described as attribution of selfhood. A closer examination of the above examples reveals that they provide evidence for social and mind attribution, for anthropomorphization, as well as for perception of intentions, agency, cognition, and consciousness or sentience. However, it is noteworthy that the authors of these examples rarely referred to the term “self” themselves. The review by Thellman et al. (2022) also found that most research refers to mental state attribution or anthropomorphism. One may argue that anthropomorphism is another suitable candidate when considering the terminology, as it covers the attribution of similar dimensions as the naïve self. However, we do not claim that the self has to be anthropomorphic. Similarly, Thellman and colleagues discuss that while anthropomorphism is a broader concept than mental state attribution, as it encompasses both mental and nonmental states, the concept is limited by its assumption that mental states are uniquely human (which is not the case, e.g., for the experience of pain). We concur that “selfhood” is just one term in this interchangeable series. The concept of self may overlap with concepts such as mind or anthropomorphism, yet it is broader than mind, more inclusive than anthropomorphism and scientifically more precise than lay conceptions such as the “soul.” Still, as we will explain further in the Discussion, we view the term self simply as an umbrella term that both scientists and laypeople alike have been unable to reach a consensus upon. Thus, we believe settling on the term self opens up the discussion to the reality that the concept has substantial ambiguity. Moreover, the present paper focuses on its use by laypeople in describing other agents and here the concept may change over time and between different contexts or between different people. This can be illustrated by the example of the tomato, where the attribution differs between laypeople and scientists. Botanists will classify the produce as fruit (see, e.g., Davies, Hobson, & McGlasson, 1981), while most laypeople will probably say that it is a vegetable. So, clearly the concept of a tomato is not the same for everybody, scientist might use cues such as the position of the seeds and laypeople might focus on the taste. However, to identify an object as a tomato, they will probably both make use of cues such as the red color and round shape—showing that external cues exist that are used to attribute this concept of a tomato. Heider (1958a) suggested that naïve concepts, like that of selfhood, are developed to make sense of ambiguous perceptual information, an account he shares with Brunswik (1955). According to Brunswik's lens model, perceived events generate proximal cues that observers integrate to derive the best‐possible interpretation (i.e., judgment). Both Heider and Brunswick assume that observers integrate various perceptual cues into the perception of a meaningful event, such as the perception of a rectangle “chasing” a “frightened” circle in the (aforementioned) Heider and Simmel film. With his attribution theory, Heider did not construe an explicit theory of the self, but when applied to the perception of selfhood, this means that attribution of a naïve self‐concept might rely on particular proximal cues provided by particular characteristics of the observed agent's behavior. Naïve observers then take these cues as evidence that the agent does or does not “possess” some kind of self. If so, it should be possible to identify the perceivable cues that people are making use of to derive the judgment that some observed agent has a self, independently of how this concept of a self (or sociality, mind, anthropomorphism, etc.) is defined specifically. Accordingly, the focus of our present study is not the attempt to explain what selfhood is, rather, it is to determine what behavioral cues have to be perceived in order to start attributing naïve concepts of selfhood to another being—be that a human or nonhuman agent.

More specifically, we considered five possible characteristics that have been championed as key cues in eliciting selfhood‐attribution in the literature. First, we considered *causality*, which we manipulated in Experiment 1. In the previously mentioned study by Heider and Simmel (1944), most participants described the geometric shapes as animate beings or even as acting people with intentions and motivations, which suggests that they attributed selfhood to these agents. In their descriptions of the stimuli, they often referred to contact and interactions between the shapes, suggesting that the ability to interact with and impact one's environment might be a relevant cue in the attribution of intention and agency to other agents. Moreover, Heider (1958b) argued in his attribution theory that causality is a crucial factor in differentiating between internal or external explanations of behavior. He posits that in everyday interaction it is of great relevance, for instance, if an action was caused by situational factors or other people. Thus, causal behavior, that indicates the ability to impact the environment or other agents, will be considered as a critical characteristic relevant to selfhood‐attribution. The second cue that we considered was *equifinality*, which we manipulated in Experiment 2. In his examination of how people perceive intention or motivation (and thus agency) in other agents, Heider (1958a) identified equifinality—the ability to achieve a goal via different means—as a critical requirement. He argued that actions are identified as intentional when behavior is perceived as flexible in achieving a goal, indicating a capability to adapt, and as motivated when behavior is persistent toward that goal. If equifinality is a requirement for intentions and motivations, it is likely to be relevant in the attribution of agency and as such a potential core characteristic in the attribution of selfhood. Third, we considered *behavioral efficiency*, which we manipulated in Experiment 3. Research in developmental psychology has shown that infants by the age of 12 months can already ascribe intention to actions but only when these are efficient (Gergely & Csibra, 2003). This shows that behavioral efficiency is critical in the attribution of agency and thereby could also be an important characteristic for selfhood‐attribution. Our fourth cue was *learning sensitivity* (i.e., the consideration of one's past experience), which we manipulated in Experiment 4. In an investigation of how humans develop to become intentional agents, Verschoor, Weidema, Biro, and Hommel (2010) have argued that the acquisition of action‐effect associations is a crucial part in the development of goal‐directedness. They found that infants start to spontaneously learn action‐effect associations already at the age of 9 months and show a preference to repeat actions that result in a previously associated event as compared to action‐independent events—replicating a similar finding for adults (Elsner & Hommel, 2001). Intentionality, in this sense, can be understood as the planning of goal‐directed actions, which is facilitated by acquired knowledge about previously experienced actions. Thus, learning sensitivity can be considered a potential core characteristic in attributing intentionality and selfhood. The fifth and final cue that we considered in the present study, and which we manipulated in Experiment 5, was *context sensitivity*. Humans excel at the ability to adjust their actions to the current environment, which is critical for cognitive control, the general ability to plan actions (Miller & Cohen, 2001). Context sensitivity is a requirement for good cognitive control, since the more complex information can be filtered by an agent, the more options for action become available to that agent. Since this is a relevant competency in showing agency, context sensitivity can also be considered an important characteristic for attributing a self to other agents.

### 1.2 Aim of the present study

To summarize, in the present paper, we follow Heider's (1958a) attribution theory in our investigation of how people perceive selfhood in other agents. While this is not an explicit theory of the self, it focuses on how people perceive others' action based on their assumed abilities, intentions, or agency (concepts central to many concrete theories of the self, as described above). It has to be stressed that the construct we are investigating is not “the self,” rather, it is the idea of a self that most people have in everyday life. There may be some hierarchy between different (sub)constructs such as “mind,” “agency,” or “higher cognitive functions,” but this can be different for each individual and they are unlikely to have a scientific and fine‐grained definition. As such, we do not propose a definite definition of what selfhood is, we see it as a highly subjective concept. This does not imply that such a thing as an objective self does not exist, but, on the other hand, the subjective experience of a sense of selfhood and the attribution of this concept also do not imply that it has to exist. To still attempt capturing the aspects of selfhood, we decided on two established (i.e., widely used across fields) questionnaires, the Mind Attribution Scale (MAS) (Bigman & Gray, 2018) and the Godspeed Scale (GS) (Bartneck, Kulić, Croft, & Zoghbi, 2009). This selection enabled a broad measurement of key aspects in various definitions of a self, whereas the MAS measured attribution of agency and experience, the GS included concepts like animacy, anthropomorphism, and perceived intelligence. This way, selfhood was operationalized as a sort of umbrella term encompassing facets that not in all individuals may be considered relevant but that between individuals are all likely to be relevant. We propose, similar to Brunswik (1955), that these attributions are elicited by the perception of behavioral cues, specifically, for the characteristics causality, equifinality, efficiency, learning, and context sensitivity.

The logic of our experiments was simple: in each, we presented participants with five short videos showing an agent who either exhibited the manipulated cue for one of the characteristics (C+) and with another five short videos showing an agent who did not exhibit this cue (C–). The videos of one of these agents were always presented on the left side of the screen and the videos of the other agent always on the right side (counterbalanced across participants). The videos were shown sequentially, alternating between C+ and C– stimuli. Thereafter, participants were to fill in questionnaires on the online platform that assessed, first, which characteristics they perceived, and second, the degree to which participants attributed selfhood^1^ to the two agents. If they would attribute significantly more selfhood to C+ than to C–, we concluded that the manipulated cue would be relevant for the attribution of selfhood. Investigating the characteristics in separate experiments allowed us to test both whether the manipulation was successful and whether it would only affect the perception of the corresponding characteristic (or if, e.g., introducing efficiency would also increase context sensitivity).

An important challenge that our study needed to meet was the choice of agents to whom participants would attribute a self in various degrees. On the one hand, overly simplified stimuli may not render the manipulation of behavioral cues very plausible, which might limit the willingness of participants to attribute a self in the first place. On the other hand, however, choosing agents that are too human‐like might induce a ceiling effect, so that our manipulations might be too subtle to show an effect. Indeed, the use of overly anthropomorphic agents has been argued to create various kinds of artifacts and confounds, and at the same time, they are used in many studies investigating attribution of mental states to robots (Thellman et al., 2022). Perceiving an agent as human‐like may already incline toward attributing more of a self, by adopting the intentional stance (see, Dennett, 1989), which is more likely with anthropomorphic robots (Marchesi et al., 2019; Perez‐Osorio & Wykowska, 2020). With the rise of humanoid robots in society (e.g., in museums, shops, education, medical contexts), identifying an agent as a robot may trigger intentional stance‐related associations even if the robot lacks a human‐like appearance. For example, Dubal et al. (2011) demonstrated that early brain potentials (P1, N170) to a smiling nonhumanoid robot were similar to that of photos of a smiling person. There is also evidence suggesting that interactions between a nonhumanoid robot and a humanoid robot led to increased anthropomorphism of the nonhumanoid robot (Ueno, Hayashi, & Mizuuchi, 2019). Accordingly, we sought to avoid any resemblance to the human form in our agents. We, therefore, chose to use modified “Duckiebot” robots (Paull et al., 2017) that, on the one hand, are sufficiently mobile to allow manipulating our five cues but, on the other hand, look much more like a vehicle than like a human—it is a platform that consists basically of a box with wheels but no further features, similar to the Braitenberg (1986) vehicles.^2^ To avoid priming participants in our instructions by using language that would suggest sentience, we consistently referred to the objects as “entities” or “the one marked with a triangle/square” rather than referring to them as robots. To further reduce potential confounds, the robots were presented in an impoverished environment, and we tried to manipulate the cues in the simplest way possible. Our key question was whether the perception of the five cues that we manipulated would significantly increase the attribution of selfhood to the respective agent or, if not, which of these cues would be most potent in doing so. We aimed at investigating this in a minimal setup to avoid as many confounds as possible (like the anthropomorphism of the robot).

## 2 General methods

### 2.1 Participants

Participants were recruited from the UK via Prolific (http://www.prolific.com) for online testing. Recruitment continued until 80 valid data sets were collected for each of the five experiments. The demographic data are presented per experiment in Table 1. In the initial experiment, participants received £6 for completing the study. As the experiments were considerably shorter than anticipated (depending on the experiment, the median duration was 11–17 min), the reward for subsequent experiments was adjusted to £4. Participants were excluded from the analysis if they reported considerable technical difficulties (e.g., stuttering of the videos, which resulted in the exclusion of *n* = 4 or 1% of the total number of recruited participants) or if they failed the attention check (as described in the *Procedure* section below, which resulted in the exclusion of *n* = 24 or 5% of all recruited participants). Participants were rejected prior to study participation when it was detected that they were using browser incompatible with the experimental software (amounting to *n* = 15 rejections or 3% of total number of recruited participants).

**Table 1:** Demographic overview of participants per experiment

Experiment	$n_{total}$	$n_{included}$	M Age	SD Age	% Female	% Male
Exp. 1	88	80	37.05	11.49	58	41
Exp. 2	85	80	36.72	12.33	34	66
Exp. 3	89	80	36.94	11.66	52	48
Exp. 4	93	80	38.44	13.12	48	52
Exp. 5	88	80	39.56	13.42	49	51

*Note*. Mean age and percentages of female and male participants refers only to the included participants. Options for gender were “female,” “male,” or “prefer not to say,” percentages, therefore, might not add up to 100.

### 2.2 Material

#### 2.2.1 Stimuli

Stimuli consisted of videos captured using a Panasonic HC‐V380EG‐K camera in our laboratory. The videos show a “Duckie Mobile Bot” (Paull et al., 2017) robot identified by either a white triangle or by a white square seen from above at an angle. We also utilized white cardboard cubes as obstacles in all experiments. Experiment 5 additionally included black cubes of the same size (see Appendix A for a visualization of example stimuli). The robots were remotely controlled by a human to cue either the presence (C+ or critical) or absence (C– or control condition) of a critical characteristic. Each experiment presented an equal number of stimuli for C+ and C–. Additionally, there was always a pair of a video showing the C+ and a video showing the C– robot in the same setup with the same duration, but between pairs, the video duration was not the same in all of the experiments. The stimuli and other material can be accessed through the Open Science Framework (OSF) (Foster & Deardorff, 2017) project (https://osf.io/b9cnr/).

#### 2.2.2 Implementation

The study was run as in‐browser experiments that participants could participate in from home. The experiments were programmed with jsPsych 7.3 (Leeuw et al., 2023) and hosted on a university server with JATOS 3.7.4 (Lange, Kühn, & Filevich, 2015).

### 2.3 Procedure

The experiment commenced with a consent form and instructions (with approval from Constructor University's ethics committee). The experiment then continued with the presentation of stimuli. C+ and C– stimuli were presented in alternation. The videos of one condition were consistently displayed either on the left or the right of the window, while the mapping of condition and alignment was systematically counterbalanced. Following the stimulus presentation, participants completed several questionnaires in which they rated each robot separately. At the end of each experiment, participants were debriefed, asked what they thought the research question was, and whether they experienced any technical difficulties with the study. Those participants who reported major problems (e.g., faulty stimulus presentation) were removed from the analysis (resulting in *n* = 4 exclusions, or 1% of all recruited participants).

The study employed a manipulation check scale (MCS) with three items for each characteristic of interest.^3^ For example, for the characteristic causality, the items “It was able to affect its environment,” “It seemed to intentionally interact with its environment,” and “It appeared to desire to modify its environment” were used. The items were composed to range from simple descriptions devoid of selfhood‐related states to descriptions referencing intentions similar to the answers of participants in Heider and Simmel's study (see Appendix B for the complete questionnaire).

For accessing the attribution of concepts indicating the naïve selfhood, two already established questionnaires were used: The GS (Bartneck et al., 2009), as well as the subscales agency, and experience of the MAS (Bigman & Gray, 2018). Carpinella and colleagues (2017), in reanalyzing the GS items, found only the factors anthropomorphism and perceived intelligence with a different factor structure. We additionally used their proposed factor structures for these two subscales (marked with an asterisk further on). This resulted in 10 items being used twice, because we did not want to remove these items from the GS to avoid tampering with the questionnaire's integrity as a whole, nor did we want to compromise the comparability with previous and future research utilizing the GS.

The scales were implemented as continuous scales, ranging from 0 to 100, with sliders marked only at the extremes: numbers 0 and 100 for the MCS; negative and positive items for the GS and Carpinella et al. factors; “Disagree” and “Agree” for the MAS. The change from Likert to continuous scales was made as, while the choice of scale has no effect within a test (e.g., García‐Pérez, 2024; Kuhlmann et al., 2017), all questionnaires were presented twice and having continuous sliders thus discouraged responses based on memory of prior responses. An attention check item asking if the robot was able to move supplemented the MAS. As the robots consistently moved in the videos, any participant who moved the response slider toward “Disagree” was excluded from the analysis (resulting in *n* = 24 exclusions, or 5% of all recruited participants). The MCS was completed by all participants first, and the order of GS, MAS, and Carpinella et al. factors was counterbalanced with the MAS always in the middle to ensure that the attention check was placed at the same point in the study flow.

### 2.4 Data analysis

All statistical analyses were conducted using R (Version 4.2.2., R Core Team, 2022). The analysis code is published in the OSF project. For each experiment, we first analyzed the manipulation check data to see what characteristics were perceived by the participants. Here, ratings were aggregated by participant, characteristic, and cue‐presence (present vs. absent) using the mean. In the analysis, we conducted a two‐way ANOVA with the within‐participant factors of characteristic (causality, speed, equifinality, efficiency, learning sensitivity, and context sensitivity) and cue‐presence (C+ vs. C–). If there was a significant interaction, we calculated post‐hoc paired *t*‐tests for cue‐presence grouped by characteristic.

Next, in cases of a significant interaction between cue‐presence and characteristic, we conducted a separate analysis of the selfhood‐attribution data for those experiments. For this analysis, we categorized in subscales rather than the overall questionnaires. The data from the MAS were, therefore, categorized into either agency or experience, while each rating from the GS was categorized as animacy, anthropomorphism, likability, perceived intelligence, or perceived safety, and the Carpinella et al. items were categorized as either anthropomorphism* or perceived intelligence*. Ratings were then aggregated by participant, subscale, and cue‐presence using the mean. This way, we calculated a two‐way ANOVA with subscales (agency, animacy, anthropomorphism, anthropomorphism*, experience, likeability, perceived intelligence, perceived intelligence*, and perceived safety) and cue‐presence (C+ vs. C–) as within participant factors. If there was a significant interaction, we conducted post‐hoc paired *t*‐tests for cue‐presence grouped by subscale. While we included perceived safety in the analysis, we did not consider this subscale as a relevant dimension in accessing selfhood‐attribution (the data are still presented).

In addition, stepwise linear mixed effect models have been computed. The results can be found in Appendix C.

## 3 Experiment 1: Causality

### 3.1 Method

**Fig. 1:**
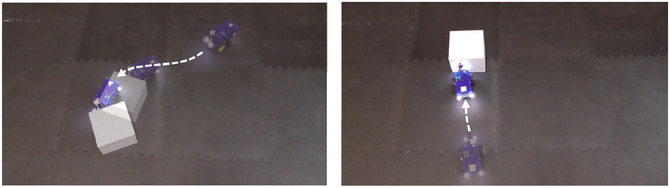
Example stimuli of C+ (left) and C– (right) for Experiment 1.

To manipulate the presence versus absence of perceived causality, two sets of five videos with a duration of 5 s were recorded. In the C+ stimuli videos, the robot collided with and moved a white cardboard cube, whereas in the C– stimuli, the robot stopped immediately after colliding with the cube and the box remained stationary. This way, the C+ robot showed a physical impact on its environment (*causing* a cube to move) that is lacking in the C– condition. See Fig. 1 for an example of both conditions visualized.

### 3.2 Results

**Fig. 2:**
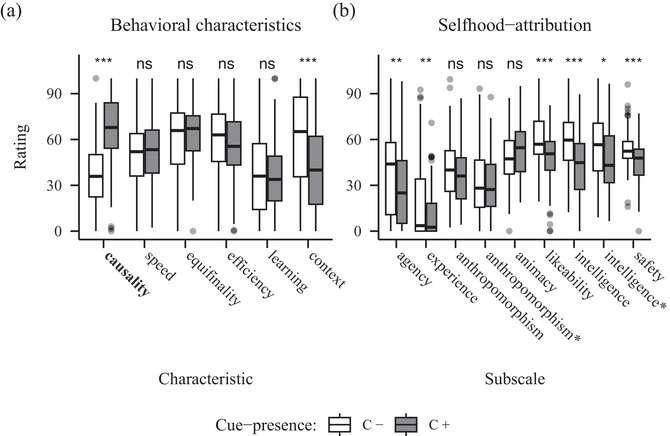
Barplots showing ratings for behavioral characteristics (A) and selfhood‐attribution (B) from Experiment 1 (causality). *Note*. Panel A: The manipulated characteristic is highlighted in bold. Panel B: Agency and experience constitute the Mind Attribution Scale. Further subscales are taken from the Godspeed Scale, while the asterisks mark the adjusted versions following Carpinella, Wyman, Perez, and Stroessner (2017). Significance codes: *p* <  .050 *, *p* <  .010 **, *p* <  .001 ***.

For the behavioral characteristics, the ANOVA revealed a significant main effect of characteristic (*F*(4.31, 340.61) = 33.61, *p* <.  001, $\eta^{2}$ = 0.10) and, more relevant, a significant interaction of characteristic and cue‐presence (*F*(3.19, 252.32) = 32.09, *p* <  .001, $\eta^{2}$ = 0.09). The post‐hoc paired *t*‐tests showed significant differences for the characteristics causality (*t*(79) = 8.95, *p* <  .001), which we aimed to manipulate and context sensitivity (*t*(79) = 3.93, *p* <  .001). The C+ robot received higher ratings in causality‐related items (*M* = 66.69, *SD* = 29.71) than the C– robot (*M* = 36.57, *SD* = 31.13), while the C– robot was rated higher on items for context sensitivity (*M* = 58.52, *SD* = 33.14) than the C+ robot (*M* = 39.95, *SD* = 30.04) (see Fig. 2A).

The analysis of the naïve selfhood‐attribution data showed significant main effects of subscale (*F*(4.52, 357.37) = 92.13, *p* <  .001, $\eta^{2}$ = 0.27]) and, importantly, of cue‐presence (*F*(1, 79) = 8.50, *p* =  .005, $\eta^{2}$ = 0.03]) and the significant interaction (*F*(4.90, 386.73) = 9.09, *p* <  .001, $\eta^{2}$ = 0.02]). Post‐hoc paired *t*‐tests further revealed significant differences for the subscales agency (*t*(79) = 2.98, *p* =  .004), experience (*t*(79) = 1.62, *p* =  .110), likeability (*t*(79) = 2.20, *p* = .031), both the original subscale perceived intelligence (*t*(79) = 4.29, *p* < .001) and the adjusted one (*t*(79) = 4.12, *p* < .001), as well as perceived safety (*t*(79) = 3.46, *p* < .001). Ratings were higher for the C– robot on all of these subscales (see Fig. 2B).

### 3.3 Discussion

Participants significantly perceived the critical robot as more causal. This was expected, because causality was manipulated in this experiment, and provides a successful manipulation check. Two other observations were not expected, however. First, the control robot was perceived as being more context sensitive. This highlights the difficulty of understanding how context is constructed in the observers' minds. The object is a context in the sense that the robot drives and is or is not aware of its environment—which can be a synonym for context. As it seems, the fact that the C+ robot kept colliding with the objects in its environment was attributed to the robot, which, in turn, provided the basis for perceiving causality. But the colliding strategy itself was apparently experienced as not overly smart and not very safe, as if the agent would be “mindlessly” bumping into any available obstacle. This is at least suggested by the second unexpected result, namely, that many of the subscales assessing selfhood were driven by context sensitivity rather than causality. In comparison to the brute‐force strategy of the C+ robot, the more gentle (situationally aware) strategy of the C– robot was perceived to be safer and as indicating greater intelligence. Taken altogether, Experiment 1 suggests that, while causality may form the basis of meaningful behavior, the perception of causality in itself may be insufficient to attribute selfhood to an agent—after all, causality could be implemented in many ways and there might be some ways where it is sufficient. Rather, context sensitivity seems to be a more relevant cue driving the attribution of selfhood to an agent.[Fig cogs70188-fig-0008]

## 4 Experiment 2: Equifinality

### 4.1 Method

**Fig. A.2:**
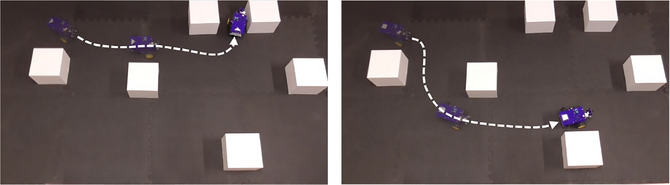
Example stimuli of C+ (left) and C– (right) for Experiment 2 (equifinality).

To manipulate perceived equifinality, two sets of five videos lasting 6–11 s each were recorded. The stimuli depicted multiple white boxes scattered about with two boxes arranged closer together than the others. In the C+ condition, the robot consistently stops its movement in the small space between the two adjacent boxes. Conversely, in the C– condition, the robot always stops alongside one of the other, distant boxes (see Fig. A.2 for an example visualization). Thus, while the C– robot chooses *arbitrary* positions as its endposition (or apparent “goal”), the C+ robot always chooses a *particular* position to stop.

**Fig. 3:**
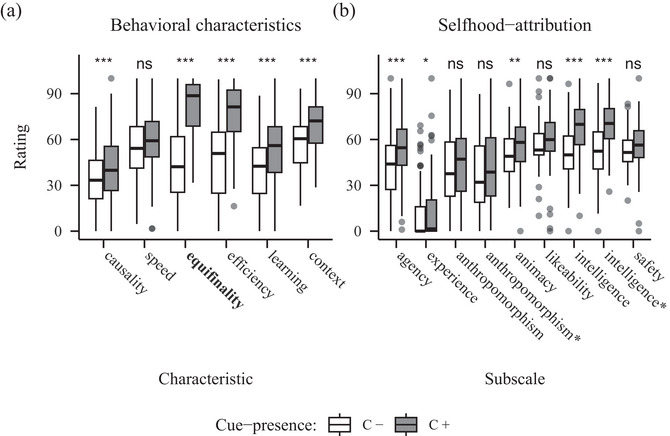
Barplots showing ratings for behavioral characteristics (A) and selfhood‐attribution (B) from Experiment 2 (equifinality). *Note*. Panel A: The manipulated characteristic is highlighted in bold. Panel B: Agency and experience constitute the Mind Attribution Scale. Further subscales are taken from the Godspeed Scale, while the asterisks mark the adjusted versions following Carpinella et al. (2017). Significance codes: *p* < .050 *, *p* < .010 **, *p* < .001 ***.

**Fig. 4:**
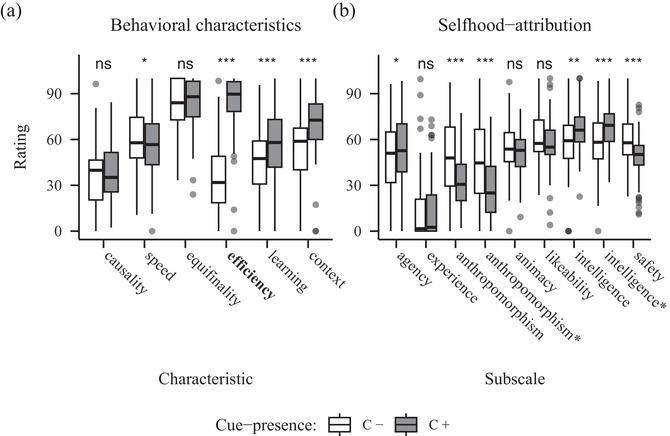
Barplots showing ratings for behavioral characteristics (A) and selfhood‐attribution (B) from Experiment 3 (behavioral efficiency). *Note*. Panel A: The manipulated characteristic is highlighted in bold. Panel B: Agency and experience constitute the Mind Attribution Scale. Further subscales are taken from the Godspeed Scale, while the asterisks mark the adjusted versions following Carpinella et al. (2017). Significance codes: *p* < .050 *, *p* < .010 **, *p* < .001 ***.

**Fig. 5:**
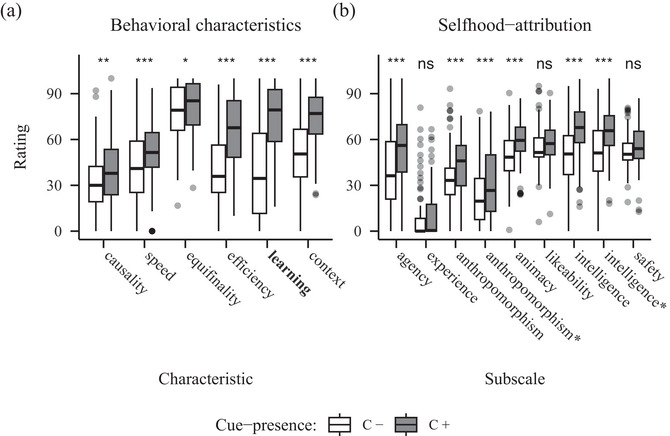
Barplots showing ratings for behavioral characteristics (A) and selfhood‐attribution (B) from Experiment 4 (learning sensitivity). *Note*. Panel A: The manipulated characteristic is highlighted in bold. Panel B: Agency and experience constitute the Mind Attribution Scale. Further subscales are taken from the Godspeed Scale, while the asterisks mark the adjusted versions following Carpinella et al. (2017). Significance codes: *p* < .050 *, *p* < .010 **, *p* < .001 ***.

**Fig. 6:**
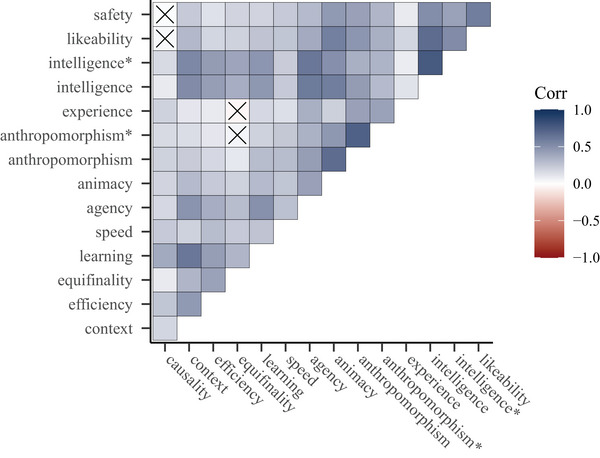
Correlation plot across all experiments. *Note*. Crossed out correlations are not significant (*p* >= .050).

**Fig. 7:**
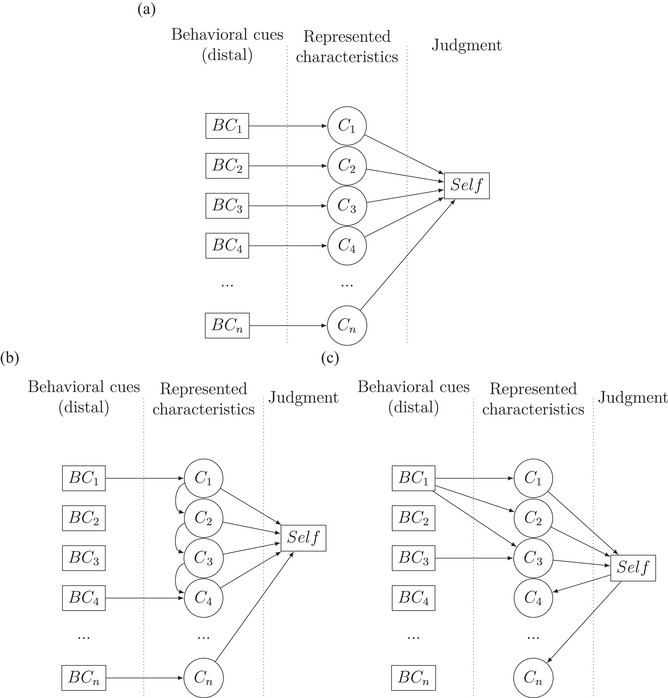
Toward a Pars‐Pro‐Toto account of selfhood‐attribution. *Note*. Panel A: The originally assumed model of naïve selfhood‐attribution. Before the data collection, we expected a 1:1 relationship between distal cues (BC) and represented characteristics (C). Panel B: Model focusing on internal overgeneralization. One perspective on our observations is that the characteristics' representations may prime each other, allowing for the activation of characteristics that are missing distal cues. Panel C: Alternative model focusing on semantic overlap between concepts underlying possible cues. This perspective highlights the possibility that external concepts such as the investigated characteristics are less organized and defined than we have considered.

### 4.2 Results

The ANOVA of the behavioral characteristics data showed significant main effects of characteristic (*F*(3.96, 313.03) = 50.96, *p* < .001, $\eta^{2}$ = 0.18]) and cue‐presence (*F*(1, 79) = 62.66, *p* < .001, $\eta^{2}$ = 0.14]) in addition to a significant interaction (*F*(3.59, 283.61) = 28.54, *p* < .001, $\eta^{2}$ = 0.07]). Post‐hoc paired *t*‐tests revealed significant differences in ratings of all characteristics except speed (see Table 2), with ratings higher for the C+ robot in all of those characteristics (see Fig. 3A).

**Table 2:** Results of the post‐hoc paired *t*‐tests for the manipulation check of Experiment 2

Characteristic	t	df	p
causality	4.46	79	< .001
speed	1.34	79	.185
equifinality	9.29	79	< .001
efficiency	7.00	79	< .001
learning	4.51	79	< .001
context	5.32	79	< .001

Next, the ANOVA of the naïve selfhood‐attribution data also revealed both significant main effects of subscale (*F*(4.42, 349.17) = 132.22, *p* < .001, $\eta^{2}$ = 0.36]) and, importantly, cue‐presence (*F*(1, 79) = 14.95, *p* < .001, $\eta^{2}$ = 0.04]), as well as a significant interaction (*F*(3.85, 303.87) = 7.60, *p* < .001, $\eta^{2}$ = 0.02]). The post‐hoc paired *t*‐tests showed significant differences in the subscales agency (*t*(79) = 4.22, *p* < .001), animacy (*t*(79) = 2.01, *p* = .048), experience (*t*(79) = 3.18, *p* = .002) and both the original subscale perceived intelligence (*t*(79) = 1.53, *p* = .131) and the adjusted one (*t*(79) = 5.06, *p* < .001); all ratings are higher for the C+ robot (see Fig. 3B).

### 4.3 Discussion

This time, participants rated the critical robot significantly higher on all characteristics except speed. Hence, they perceived the robot as exhibiting causal behavior, equifinality, behavioral efficiency, learning sensitivity, and context sensitive behavior. Regarding selfhood‐attribution, the C+ robot received significantly higher ratings for the important subscales of agency, animacy, experience, and perceived intelligence. Taken altogether, this result pattern can be looked at from two angles. On the one hand, the findings suggest that our manipulation worked well, and that this manipulation resulted in the naïve attribution of selfhood to the agent which was perceived with the critical cue—equifinality. This, in turn, indicates that equifinality is a potent perceptual cue in and eliciting selfhood‐attributions, which is what the present study was looking for.

On the other hand, however, the overall result pattern suggests that our original approach was probably too simplistic. Two observations support this suspicion. First, our manipulation was not only successful in increasing the perception of the C+ robot as exhibiting equifinality. It also significantly increased most other ratings of the MCS, which indicates that our manipulation did way more than we expected it to do. Second, and relatedly, our attempt to increase equifinality was as successful in increasing the perception of causality as was Experiment 1, only that here causality was positively correlated with context sensitivity (*r* = .280, *p* < .001). In fact, all scales of the MCS were significantly correlated (all *p*‐values <= .009) with moderate coefficients accounting for 40% and strong coefficients accounting for 27% of all correlation coefficients.

In view of these findings, our original hypothesis might have been too naïve by implicitly assuming independence between the possible cues and independent connections between cues and the selfhood concept. As it seems, manipulations targeting the perception of a particular cue can be less efficient in driving judgments related to this cue than manipulations targeting another selfhood cue. One may, of course, argue that our manipulations simply did not work as expected. It may be that the manipulation of equifinality in Experiment 2 actually was a better, or more appropriate manipulation implying causality than our attempted manipulation of this cue in Experiment 1. However, given that both manipulations significantly affected causality, and the manipulation of equifinality was driving more other scales than the manipulation of causality in Experiment 1, this possibility fails to fully account for the observations. Moreover, a closer look at the stimulus also fails to provide support for an account in terms of unsuccessful manipulation. We used white cubes as obstacles, two of which were closer to each other than the rest. The C+ robot exhibiting the critical cue consistently moved to the space between these two cubes and halted its movement there, while the C– robot in the control condition stopped next to one of the other cubes. If participants attributed goal directness to the robots as we intended, then it could indeed easily follow that they perceived the C+ robot as being more context sensitive because it “notices” that two cubes are closer to each other compared to the control robot, which seems oblivious to this or at least suggests with its behavior to ignore it. In specific, the critical robot shows behavior indicating to act based on the ability to “notice” when two cubes are close to each other, which is reflected in context sensitivity items like “It appeared to pay attention to its environment” (see Appendix B for a complete list of items used in the MCS). In contrast, when it comes to other characteristics, such as causality (assessed through phrases like “It was able to affect its environment”), the stimuli do not provide any distinctive information about the two robots. Both moved without touching any obstacles, and none of the obstacles moved on their own. Therefore, there is no perceptual basis to presume that the robots differ in how causal their behavior is. This, in turn, suggests that the perception of causality was apparently not driven by the processing of sensory information. Rather, it seems that the attribution of some characteristics can be driven by internal representations of other characteristics. In other words, representations of characteristics related to selfhood can become activated through both sensory evidence from the event one is observing (i.e., through direct perception) and a more “lateral” priming through representations of other selfhood‐related characteristics.

We will suggest a detailed model of this possibility in the *General Discussion*, but would like to point out already that our result pattern is reminiscent to what has been referred to as Halo effect. First reported by Thorndike (1920), this effect suggests that observers tend to overestimate correlations between seemingly related (but logically and empirically unrelated) feature dimensions. For instance, they consider people scoring high on a particular positive trait, such as attractiveness, as also likely to score high on other positive (but logically and empirically unrelated) traits, like intelligence (see, Forgas & Laham, 2016). While for selfhood‐attribution, the behavioral cues are not necessarily perceived as positive, we found a similar “illusory correlation.” That is, our findings are similar in the sense that when participants perceive an agent as context sensitive, say, they also perceive it as more causal, efficient, and learning sensitive. Hence, perceiving one selfhood feature to an agent is likely to trigger the attribution of other selfhood features to this agent—regardless of the available sensory evidence.

## 5 Experiment 3: Behavioral efficiency

### 5.1 Method

**Fig. A.3:**
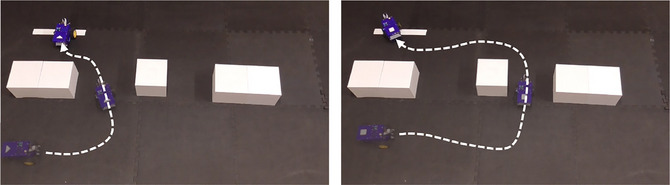
Example stimuli of C+ (left) and C– (right) for Experiment 3 (behavioral efficiency).

To manipulate perceived behavioral efficiency, two sets of five videos, each 6–8 s in length, were created. The stimulus layout features a white tape marking the end point of the robots' movement on the screen, along with several white boxes organized in such a way that there are short and longer paths leading to the apparent goal position. In the C+ condition, the robot always moves along the shorter path, while in the C– condition, the robot always moves along the longer path. Both robots come to a stop at the apparent goal position (see Fig. A.3 for a visualization of an example). In other words, the C+ robot reaches the goal position in less time and with less movement, behaving more efficient than the C– robot.

### 5.2 Results

The analysis of the behavioral characteristic data showed significant main effects of characteristic (*F*(3.85, 304.49) = 92.10, *p* < .001, $\eta^{2}$ = 0.31]) and cue‐presence (*F*(1, 79) = 61.98, *p* < .001, $\eta^{2}$ = 0.09]), and more interestingly, a significant interaction (*F*(3.83, 302.47) = 64.60, *p* < .001, $\eta^{2}$ = 0.16]). The post‐hoc tests revealed significant differences for the characteristics speed (*t*(79) = 2.15, *p* = .034), behavioral efficiency (*t*(79) = 12.65, *p* < .001), learning sensitivity (*t*(79) = 4.08, *p* < .001), and context sensitivity (*t*(79) = 5.70, *p* < .001). The C+ robot was rated higher on items related to all of those characteristics except speed, where the C– robot received higher ratings (see Fig. 4A).

The ANOVA for the naïve selfhood‐attribution data revealed a significant main effect of subscale (*F*(4.53, 358.04) = 129.84, *p* < .001, $\eta^{2}$ = 0.38]). While there was no significant main effect of cue‐presence, interestingly, we observed a significant interaction of subscale and cue‐presence (*F*(4.72, 373.02) = 23.60, *p* < .001, $\eta^{2}$ = 0.06]). The post‐hoc tests showed significant differences for the subscales agency (*t*(79) = 2.30, *p* = .024), both original anthropomorphism (*t*(79) = 5.15, *p* < .001) and adjusted (*t*(79) = 4.90, *p* < .001), also both original perceived intelligence (*t*(79) = 1.66, *p* = .100) and adjusted (*t*(79) = 2.77, *p* = .007), as well as perceived safety (*t*(79) = 4.51, *p* < .001). Ratings of the agency subscale were higher for the C+ robot (*M* = 52.96, *SD* = 31.30) compared to the C– robot (*M* = 48.36, *SD* = 30.78); on the anthropomorphism subscales, ratings were higher for the C– robot on both the original subscale (*M* = 48.54, *SD* = 32.11) and the adjusted (*M* = 46.22, *SD* = 32.60), than the C+ robot on the original (*M* = 33.12, *SD* = 27.50) or adjusted version (*M* = 27.15, *SD* = 26.75); ratings for the subscales perceived intelligence were higher for the C+ robot on the original (*M* = 67.11, *SD* = 19.46) and adjusted version (*M* = 68.11, *SD* = 20.44) compared to the C– robot both for the original (*M* = 58.74, *SD* = 22.91) and adjusted subscale (*M* = 58.99, *SD* = 23.51); finally, ratings for the subscale perceived safety were higher for the C– (*M* = 59.72, *SD* = 23.91) robot than for the C+ robot (*M* = 48.84, *SD* = 23.74) (see Fig. 4B).

### 5.3 Discussion

A similar pattern emerged as in Experiment 2. Participants rated the C+ robot as significantly more context sensitive, equifinal, efficient, and learning sensitive than the C– robot. Surprisingly, the control robot was perceived to move at a more human‐like speed compared to the C+ robot. While the C– robot was rated significantly higher in terms of its anthropomorphism and perceived safety, the C+ robot was perceived as significantly more likely to have agency and to be intelligent. The results further strengthen the interpretation that, as soon as an agent is perceived as having a self for some reason (i.e., as suggested by one cue), the perception of its behavior works similar to the Halo effect (in the form of an illusory correlation) as the perception of characteristics is extended to other uncued characteristics. In this experiment, speed was the only exception. The C– robot was perceived to move at a more human‐like pace. Therefore, it is not unexpected that the robot received a higher rating on the anthropomorphism subscale but, as most of the other experiments show, this subscale was not very sensitive to other aspects of selfhood.

## 6 Experiment 4: Learning sensitivity

### 6.1 Method

**Fig. A.4:**
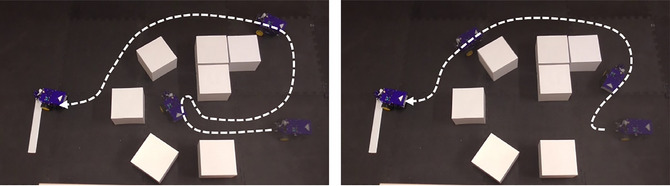
Example stimulus set of the C+ condition for Experiment 4 (learning sensitivity). *Note*. For this experiment, an individual stimulus consisted of two videos that were shown directly one after the other. On the left, a frame from the first video is shown, and on the right, a frame from the second video of a C+ stimulus. In this condition, the robot alters its behavior based on what it experienced in the first video, while the C– robot always presents the same behavior in both videos.

To manipulate perceived learning sensitivity, two sets of five pairs of videos were produced, each lasting 12–22 s. For each condition, five sets of two videos were recorded, with the second video of each set always shown immediately after the first video. The setup consisted of white cubes serving as obstacles and a white line as a target. The cubes were positioned in a manner that would make it ambiguous to the robot which path was the shortest or made one path look shorter but was blocked outside the robot's field of view. In the event of an unclear shortest path, the robot opted for the longer route in both conditions, in the first video. However, in the subsequent video, the C+ robot followed the shorter path revealed in the first video, while the C– robot continued to move along the longer path. If the seemingly shorter path was obstructed in the first video, the robot proceeded along that path until it was impeded before returning and taking the alternative route, for both conditions. In the second video, the C+ robot moved directly along the path toward the goal. In contrast, the C– robot initially attempted the seemingly direct path again (see Fig. A.4 for a visualization of an example). Thus, in contrast to the C– robot, the C+ robot shows a transfer of “experience” or apparent memory of its surrounding.

### 6.2 Results

The ANOVA for the behavioral characteristics data showed significant main effects of characteristic (*F*(3.91, 308.62) = 79.82, *p* < .001, $\eta^{2}$ = 0.27]) and cue‐presence (*F*(1, 79) = 62.12, *p* < .001, $\eta^{2}$ = 0.13]), and importantly also a significant interaction (*F*(3.17, 250.38) = 23.55, *p* < .001, $\eta^{2}$ = 0.06]). The post‐hoc paired *t*‐tests revealed significant differences for all characteristics (see Table 3), with ratings always higher for the C+ robot (see Fig. 5A).

**Table 3:** Results of the post‐hoc paired *t*‐tests for the behavioral characteristics of Experiment 4

Characteristic	t	df	p
causality	2.77	79	.007
speed	3.87	79	< .001
equifinality	2.41	79	.018
efficiency	6.52	79	< .001
learning	8.15	79	< .001
context	6.75	79	< .001

Next, the ANOVA for the naïve selfhood‐attribution data revealed significant main effects of subscale (*F*(4.54, 358.39) = 165.39, *p* < .001, $\eta^{2}$ = 0.42]) and, more relevantly, cue‐presence (*F*(1, 79) = 26.13, *p* < .001, $\eta^{2}$ = 0.05]) and the interaction (*F*(4.79, 378.32) = 10.72, *p* < .001, $\eta^{2}$ = 0.02]). The post‐hoc *t*‐tests showed significant differences for the subscales agency (*t*(79) = 5.77, *p* < .001), animacy (*t*(79) = 1.98, *p* = .051), both original anthropomorphism (*t*(79) = 3.77, *p* < .001) and adjusted (*t*(79) = 3.89, *p* < .001), as well as both the original perceived intelligence (*t*(79) = 1.34, *p* = .184) and its adjusted version (*t*(79) = 5.37, *p* < .001); with ratings always higher for the C+ robot (see Fig. 5B).

### 6.3 Discussion

Again, we observed a pattern akin to the previous experiments. The C+ robot was perceived as expressing all traits significantly more strongly than the C– robot, and also received significantly higher ratings for agency, animacy, anthropomorphism, and perceived intelligence. Therefore, this experiment replicates the Halo‐like effect of attributing behavioral characteristics to robots that participants are more likely to attribute a naïve self to.

## 7 Experiment 5: Context sensitivity

### 7.1 Method

**Fig. A.5:**
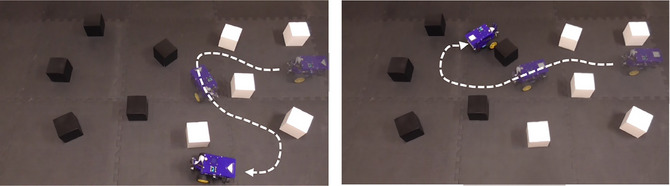
Example stimuli of C+ (left) and C– (right) for Experiment 5 (context sensitivity).

To manipulate perceived context sensitivity, two sets of five videos, each lasting 9–12 s, were recorded. The videos depicted white and black boxes arranged in separate areas consisting of only one type of cube. The C+ robot moved exclusively within areas in which it started, for example, if it started next to white cubes, it moved only within the area of white cubes, while the C– robot always moved through both areas (see Fig. A.5 for the visualization of an example). Therefore, the C+ robot showed apparent “awareness” of the surroundings (staying within one area) and the C– robot did not show this capability.

### 7.2 Results

The ANOVA of the behavioral characteristics data only showed a significant main effect of characteristic (*F*(4.12, 325.28) = 34.21, *p* < .001, $\eta^{2}$ = 0.15]). Since the interaction was not significant (*F*(3.35, 264.38) = 2.43, *p* = .059, $\eta^{2}$ = 0.00]), no further analysis, specifically that of the selfhood‐attribution data, was conducted.

### 7.3 Discussion

While in all of the previous experiments participants rated the two robots different in regard to context sensitivity, they did not in this experiment. Further, we observed no notable distinctions among the robots on any of the characteristics. It is difficult to draw any concrete conclusions based solely on this outcome. However, when considering the interpretation of Experiments 2 through 4, it is possible that context sensitivity alone may not be adequate in inducing this Halo‐like effect. This interpretation is supported by the results of the first experiment. In that study, participants perceived the C– robot as more context sensitive and rated it higher on critical selfhood subscales, but not on other characteristics. Therefore, it appears that while context sensitivity may be relevant to the attribution of selfhood, it is unlikely to be critical for the Halo effect like perception of selfhood‐related characteristics.

## 8 Joint analysis

As an exploration of the results from Experiments 2 through 4, we calculated Pearson's correlation coefficients for ratings of the characteristics and selfhood‐attribution subscales across all experiments. This analysis showed that almost all characteristics and subscales have significant, positive correlations (only four coefficients, corresponding to 4% of the total amount, have a *p*‐value of .05 or bigger), with moderate coefficients accounting for 32% and strong coefficients accounting for 19% of all coefficients (see Fig. 6 for visual representation). Note that, unsurprisingly, the two highest coefficients are the correlations between the original factors of anthropomorphism (*r* = .731, *p* < .001) and perceived intelligence (*r* = .775, *p* < .001), with their slightly adjusted versions (marked with an asterisk in Fig. 6) after Carpinella et al. (2017).

Most characteristics and selfhood‐attribution subscales were significantly correlated, with the majority of correlation coefficients being moderate to strong. This suggests that, even in the absence of evidence for particular selfhood‐related behavioral characteristics, given that any single characteristic is perceived, this drives a general tendency of attributing selfhood‐related characteristics, as well as selfhood itself.

Additionally, for each experiment and condition, we calculated Cronbach's alpha for the subscales of the selfhood‐attribution data. Results indicate that in 70% of the instances, Cronbach's alpha is acceptable or better (>0.70), suggesting that the analysis on a subscale level rather than questionnaire level is still adequate.

## 9 General discussion

The aim of this study was to better understand the role of perceiving simple behavioral cues in the subjective attribution of selfhood to other (nonhuman) agents. We examined five characteristics that have been considered as potential core cues of naïve concept of selfhood, namely, causality (Experiment 1), equifinality (Experiment 2), behavioral efficiency (Experiment 3), learning sensitivity (Experiment 4), and context sensitivity (Experiment 5). This was done by having participants rate the behavior of small nonhumanoid, vehicular‐like robots on an MCS, as well as established self‐related questionnaires. We manipulated the robot's behavior to suggest either the presence (C+) or absence (C–) of one of the potential core characteristics.

In the first experiment, participants did perceive the C+ robot in the critical condition to show significantly more causal behavior (as we anticipated), but to our surprise, the C– robot in the control condition was perceived as significantly more context sensitive than the C+ robot. Moreover, the C– robot received significantly higher scores than the C+ robot on a majority of the selfhood‐related subscales agency and experience, as well as on the subscale perceived safety. Hence, even though causality as a characteristic might form the foundation of meaningful behavior, it seems to be less relevant as a cue in attribution of selfhood than context sensitivity.

In Experiments 2–4, we observed another unexpected result. Participants perceived selfhood relevant characteristics in an agent even though these characteristics were not evident from its behavior. At first sight, our findings seem paradoxical: On the one hand, even very important cues like causality or context sensitivity are insufficient to trigger the attribution of selfhood. On the other hand, cues that seem less relevant are able to drive the perception of causality and context ability, and eventually the subjective attribution of selfhood. How is this possible?

### 9.1 Theoretical implications

Our original hypothesis implicitly assumed unique connections between the behavioral cues that agents provide to observers and internal representations of corresponding characteristics. Had this hypothesis been correct and assuming that our manipulations were successful, we should have observed 1:1 relationships between the manipulated cues, such as causality, and corresponding judgments, as assessed by the causality subscale of our MCS. Accordingly, some cues might have driven their corresponding judgment, without affecting any other judgment, which, in turn, should have driven attributed selfhood. This implicit assumption of ours could be couched in terms of a Brunswikian lens model (Brunswik, 1955) (like the one in Fig. 7A). Each behavioral cue that the behavior of an agent would reveal (BC_1_ … BC_n_) would lead to the activation of a corresponding internal representation of the respective characteristic (C_1_ … C_n_), and then some of these internal representations would converge on to some selfhood judgment. However, our findings suggest that this scenario does not quite work. For one, not all behavioral cues directly activate their corresponding representations (e.g., our manipulation of context sensitivity in Experiment 5 failed to elicit corresponding judgments of context sensitivity, even though a paradigm was clearly suited to elicit such judgments). This means that the perception of some behavioral cues do not entertain direct associations with the representations of the characteristics they inform about, as indicated by the fact that some BCs are associated with a particular C, while others are not. For another, some representations were apparently not activated by their own behavioral cues, but by the behavioral cues of other representations (e.g., providing behavioral cues about equifinality was driving the perception of causality judgments in Experiment 2). To account for this observation, we propose two alternative explanations or, given that they share a number of implications and given that they are not mutually exclusive, two versions of one alternative explanation (see Fig. 7B, C). Both of these versions share the assumption that, in contrast to our original, purely Brunswikian model (shown in Fig. 7A), there is no 1:1 relationship between behavioral cues and represented characteristics, so that the same judgment can be triggered by the perception of different behavioral cues and/or different represented characteristics.

The first version of our alternative model (Fig. 7B) deviates from our original model in two ways: (1) it gives up the assumption that each represented characteristic is necessarily driven by a unique behavioral cue; and (2) it allows represented characteristics to prime each other. This means that each represented characteristic can be activated in two different ways: directly, in the presence of a dedicated distal cue (i.e., direct perception), and indirectly, through another activated represented characteristic. This means that represented characteristics and resulting judgments can go beyond the available information by relying on previously acquired associations between represented characteristics. Accordingly, a configuration of that kind would be able to generate not only findings of the kind obtained in this study, but any kind of Halo effect (or illusory correlation)—which means that this model can account for effects that go way beyond our present observations. Applied to selfhood, this model suggests that just the perception of one cue or a combination of just a few cues, can lead to the attribution of selfhood and to a corresponding judgment. Note that such a scenario may or may not involve anything like a concept of a self. On the one hand, it might be that we implicitly acquire such a concept; however, it might be defined, and need only relatively little evidence to trigger it, which, in turn, steers our action correspondingly. On the other hand, however, the model is also consistent with the idea that all we have is a loose network of interconnected representations of characteristics—a kind of “cognitive bundle” self (c.f. Braitenberg, 1986; Hume, 1739, as discussed in the Introduction). In our scenario, this bundle self may not refer to anyone in particular, neither oneself nor to some specific other. It is just a kind of distributed concept or, say, a conceptual network that captures what people have learned to relate to selfhood.

This has indeed been claimed by Hommel (2019b), who has argued that people represent the concept of their self by means of lose networks of feature codes. Codes may contribute to representing oneself or others to the degree that these are activated in the current situation. This activation may vary over time and space, which implies that there may not be any specific, definite definition of oneself or any. Rather, different features may represent oneself and others under different circumstances (see, e.g., Banakou, Kishore, & Slater, 2018; Ma, Sellaro, & Hommel, 2019, for experimental manipulations of potential feature codes). Accordingly, it may very well be that the role of the cues that we have considered in this study may vary with the situation a perceived agent is confronted with. The role of the cues may also vary with other available agents. For instance, in the presence of other, “dumber” robots, our critical robot might need more specific cues or other cues to receive high selfhood ratings. It may also matter whether and how the robot is interacting with others, and how this interaction can be characterized. We are currently planning experiments that are investigating this issue.

Our second version of an alternative model was motivated by the possibility that the looseness of self‐representation may not or not just reflect a lack of stricter organization within the agent, that is, of the represented characteristics. Rather, it may be the external concepts that are less organized, less unique, and less well‐defined than we have considered in this study. Indeed, Danziger (1997) has pointed out that most concepts used in cognitive research are derived from everyday language, which is not well‐suited to provide clear‐cut, unique concepts for driving mechanistic research. Indeed, a closer look reveals that many concepts used in science are less well‐defined than researchers seem to assume. For instance, the concept of attention has been used in various, partly mutually incompatible ways, suggesting that we actually do not really know what attention is (Hommel et al., 2019). Worse, the inaccurate definitions of many concepts are likely to introduce substantial overlap between them, especially when considering the underlying mechanisms. For instance, concepts like emotion and action control are likely to overlap substantially with respect to the neural underpinnings, which implies, among other things, that asking how emotion and action control interact is a rather meaningless question (Hommel, 2019a). Applied to our study, and to the question of how the perception of behavioral cues are related to represented characteristics in general, these considerations imply that the distal features that we aimed to manipulate in the present study may overlap conceptually. Hence, the actual everyday life meanings of concepts like causality, equifinality, efficiency, or contextual sensitivity may not be completely distinct but may overlap. In the same way, it may be that the concept of self is much broader than we expected and includes what we assume to be independent characteristics. In model terms, this would imply on the one hand that it is actually the distal behavioral cues that in some sense overlap, so that each cue may activate more than one dedicated represented characteristics. More specifically, the same represented characteristic may be activated by various behavioral cues or, more precisely, by several features or future components that the behavioral cues entail (this possibility is expressed in Fig. 7C). Similar, activating some characteristics via one or more distal cues and thus triggering a selfhood judgment might, in turn, trigger the activation of other uncued characteristics (as indicated by the backward arrows from self to C_4_ and C_n_ in 7C) because these characteristics are simply included in people' definition of a self. Either way, this model also gives up the assumption of a 1:1 relationship between distal cues and represented characteristics and allows one behavioral cue to activate different characteristics, which also implies that the same characteristic can receive input from different behavioral cues.

As pointed out, this second model version is not entirely distinct from the first, and it is possible that an integrated model with distal cues priming various represented characteristics and at least some characteristics priming other characteristics directly turns out to be the most suitable one. Nevertheless, while the first version directs attention to the possible interactions between representations within the agent, the second version attracts attention to the reality of stimuli and their relationship to laypeople's concepts. It is possible that our stimuli and our manipulations of features have affected the perception of more than a single characteristic (as implied by Experiment 1, where the control robot by not showing cues for the ability to affect its environment signaled the ability to be sensitive to its context). Even though the logic underlying our two alternative versions differs, it will be hard to empirically distinguish between them and, as pointed out, the two versions may also be integrated into a single model. In any case, however, our findings suggest that the relationship between the perception of distal cues and represented characteristics in a Brunswikian sense will often not be 1:1, which implies that the attempt to identify unique distal triggers for particular characteristics related to selfhood is likely to be futile. Rather, a few behavioral cues will be sufficient to trigger naïve judgments of selfhood that go way beyond the actual perceived information, be it through a semantic overgeneralization of features or internal overgeneralization of represented characteristics, or both. Hence, we take just a few parts for a whole, so that both of our alternative models can be considered versions of a *Pars‐Pro‐Toto* account of selfhood‐attribution. The perception of very little distal information seems to be sufficient to trigger rather far‐reaching conceptualizations of other agents as possessing a self.

### 9.2 Practical implications

Further understanding how behavioral characteristics influence the concept of selfhood has significant implications not only for theoretical considerations, but quite concretely for designing robots and technological systems, as well as for enhancing human–robot interactions. With the advance of robots into different ares of our society, no longer limited to industry, it becomes important to consider people's attitudes toward robots (Basori et al., 2020; Corrales‐Paredes et al., 2023). By emphasizing key traits that evoke a sense of self in robotic systems—such as beliefs, attitudes, and personality—designers can foster more effective interactions, promoting trust, empathy, and collaboration (Chatzoglou et al., 2024). Simplifying selfhood into manageable traits can streamline development, making integration into various contexts more feasible. However, in some contexts like autonomously driving robots, attributing selfhood to robots may also impose challenges in human– robot interactions (Thellman & Ziemke, 2021; Ziemke, 2020). In this case, it becomes important to ensure realistic perception of robots' capabilities. Our study suggests that the selfhood‐attributions may be a product of the observer's social expectations, which is inline with Waytz' (2010) discussion of mind perception as a social construct driven both by the agent and observer. However, as the perception of the robot agent may go way beyond its actual capabilities, this highlights the additional need for better understanding of how already simple behavioral cues of an agent may elicit complex attributions in humans. Further, Bigman and colleagues (2019) argue that as robots become more autonomous, it will become more likely for people to perceive them as moral agents. In this ethical discussion, it becomes necessary to not only consider whether a technical system (be it a robot or artificial intelligence (AI)) is capable of making moral decisions but to also consider whether the human interacting with the system perceives it to be capable of making such decisions (see also Gunkel, 2018). Overall, in one way or the other, the knowledge of how humans perceive other agents based on their behavior can inform ethical guidelines for AI and robotics, ensuring alignment with societal values and incorporating this understanding can create more user‐friendly robotic systems that promote positive and safe interactions between humans and embodied artificial systems.

### 9.3 Limitations

As mentioned earlier, some results from Experiments 2 to 4 may be accounted for by nonintended specifics of the stimulus design. However, arguably, not all of the characteristics that participants perceived in the critical robot across experiments can be explained by confounds in the stimuli (as laid out in more depth in the Discussion of Experiment 2). Moreover, while there is evidence that the perception of some characteristics elicits the attribution of a cluster of characteristics, we cannot in all cases identify which exact characteristics are needed for this effect. Our findings indicate that causality and context sensitivity alone may not be enough to initiate this phenomenon (i.e., in our models, for these characteristics, there seems to be no connection between the corresponding behavioral cues and unrelated represented characteristics). However, we cannot determine the influence of other characteristics. Therefore, we cannot make a claim as to which of the two proposed model version better reflects our observations: participants may have perceived the presence of multiple features that activated various characteristics. Future studies should concentrate on thoroughly distinguishing these characteristics and the connections of their cues and representations and on distinguishing between the two proposed accounts, if needed. Although, it might very well be that people simply think about selfhood in a less organized way using concepts that are overlapping more than it is portrayed in the current literature.

While our approach frames selfhood as a naïve theory in terms of Heider (1958a), using certain items in accessing people's naïve concepts does represent a selection of dimensions. By using multiple questionnaires, we attempted to include most of the available dimensions found in the scientific discourse opening or assessment up to many theoretical perspectives. Moreover, there are other (potentially less frequently used) questionnaires covering the same or similar dimensions, our selection of established questionnaires was also done to enable comparability to studies using only any single of the instruments included here. However, one can argue that our list is not exhaustive and that an even broader measure would be needed in the future. Moreover, we do not present any evidence of what factors drive these subjective concepts: For example, there might be cultural differences not captured in our mostly “WEIRD” sample (see Henrich, Heine, & Norenzayan, 2010). This seems highly likely, considering that in the conception and attribution of agency (a core dimension of selfhood in many theories), there are variations found between cultures (Kühnen & Kitayama, 2021). Another likely candidate would be familiarity with the observed agent, that Epley et al. (2007) found to be one of three factors of anthropomorphization. These are open questions not addressed in the present work, which is only a very first step at investigating selfhood as a naïve theory.

We used a nonhumanoid robot that looks more like a vehicle than a “stereotypical” robot as stimulus. We expect the results to generalize to more stereotypical and humanoid robots, as the literature suggests anthropomorphism as another key factor in selfhood‐attribution. As our participants were recruited from the UK, we have no evidence on whether our findings would replicate with participants from cultures with less exposure to robots or advanced technology in general. We have no reason to believe that the results depend on other characteristics of the participants, materials, or context.

### 9.4 Conclusion

Our study sheds light on the factors that contribute to the attribution of selfhood—understood as an umbrella term for lay people's complex attributions of, for example, sociality, a mind, or generally anthropomorphism—to other agents and provides novel evidence for the case of nonanthropomorphic agents. We showed that the perception of simple behavioral cues of several characteristics are relevant for the naïve attribution of selfhood. Further, we found that these cues are linked in a Halo effect like cluster (i.e., an “illusory” correlation) and are thereby rarely perceived in isolation. We present two alternative Brunswikian‐style lens models of selfhood that can account for the observed effects. They can both be considered Pars‐Pro‐Toto models of selfhood‐judgment, but they emphasize different aspects of going beyond the information given: one focuses on internal overgeneralization, which can also account for other Halo‐like observations, while the other focuses on semantic overlap between the concepts underlying possible behavioral cues. In any case, our present data suggest that equifinality, behavioral efficiency, and learning sensitivity (characteristics defined distinctly in the previous literature) are either likely candidates for triggering the Halo‐like perception of cues relevant in the subjective selfhood‐attribution or have a bigger semantic overlap in the way that people perceive these characteristics than has previously been assumed. Further, we provide an addition to the evidence that these characteristics, just as causality and context sensitivity, generally play a role in the naïve selfhood‐attribution to other agents.

## Funding

This work was funded by the German Research Foundation, DFG, in the form of a grant awarded to BH, AK, and FM within the DFG Priority Program 2134 “The Active Self” (HO 1430/13‐1). The Article Processing Charge (APC) was funded by the joint publication funds of the TU Dresden, and the “Sächsische Landesbibliothek – Staats‐ und Universitätsbibliothek” (SLUB) Dresden.

## Conflict of interest

The authors declared that they had no conflicts of interest with respect to their authorship or the publication of this article.

## Ethics approval and consent to participate

Ethics approval was given by the ethics committee at Constructor University (application number 2020_03). Informed consent was obtained from all individual participants included in the study.

## Data, materials, and code availability

Data and materials (stimuli, analysis script) are accessible via the Open Science Framework project (https://osf.io/b9cnr/).

## Figures and Tables

**Fig. A.1 cogs70188-fig-0008:**
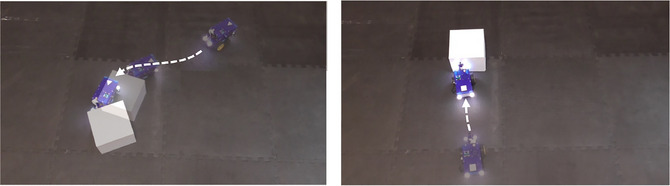
Example stimuli of C+ (left) and C– (right) for Experiment 1 (causality).

## Notes

1 Following Heider's attribution theory, we did not explicitly ask participants if the robots have a self, rather we used scales accessing the perception of personal abilities (e.g., intelligence) and agency or if participants perceived the robots to be conscious.

2 Braitenberg vehicles are simple robots equipped with two wheels connected to primitive sensors. They are designed to move autonomously in reaction to external stimuli. Varying the connection between sensors and wheels results in different behaviors of the robot, such as goal‐directed behavior even though it is a strictly mechanical agent.

3 Note that the manipulation check scale includes a cue that was not directly related to the experiments reported here: speed. The reason is that we had planned an experiment comparing human versus nonhuman speed, which due to technical and conceptual problems has not been completed so far, and piloting for which revealed that issues of absolute and relative speed are sufficiently complex to motivate a separate study that exclusively focuses on speed.

## Appendix B Manipulation check scale

### 1

1. Causality

(a) It was able to affect its environment.
(b) It seemed to intentionally interact with its environment.
(c) It appeared to desire to modify its environment.

2. Speed

(a) It appeared to move with human‐like speed.
(b) It appeared to move at walking pace.
(c) It seemed to be moving at just the same speed that I would have been moving if I had been in its place.

3. Equifinality

(a) It seemed to always move to a particular place.
(b) It seemed to have a goal when moving.
(c) It looked like it aimed to reach a specific spot.

4. Behavioral efficiency

(a) It appeared to move along the shortest pathway.
(b) It appeared to want to move efficiently.
(c) It seemed to pick the easiest way.

5. Learning sensitivity

(a) It seemed to learn from experience.
(b) It looked like it wanted to learn from its mistakes.
(c) It seemed to benefit from the experiences it made with its environment.

6. Context sensitivity

(a) It appeared to be sensitive toward signals in its environment.
(b) It seemed to pay attention to its environment.
(c) It seemed to take changes in its environment into account when deciding what to do.

## Appendix C Additional analysis

### 1

In addition to the main analysis, generalized linear mixed‐effects models have been computed for each experiment using the *lme4* R‐package. Stepwise comparisons were computed with likelihood ratio tests, comparing a null model with models only containing a single main factors, which were compared with a model containing all main factors, which was then compared to a full model also including the interaction. For the behavioral characteristics, the formula for the full model was “rating∼1+cue+characteristic+cue∗charactersistic+(1|participant)” and for the selfhood‐attribution, the full model was specified as “rating∼1+cue+subscale+cue∗subscale+(1|participant).”

Overall, the results support the main analysis, as across all experiments, except in Experiment 5, the full model best explains the data. In Experiment 5, the model comparisons, just as the main analysis, do not suggest that there is an interaction of the characteristics with the cue presence, rendering the analysis of the selfhood‐attribution irrelevant.[Table cogs70188-tbl-0004], [Table cogs70188-tbl-0005], [Table cogs70188-tbl-0006], [Table cogs70188-tbl-0007], [Table cogs70188-tbl-0008], [Table cogs70188-tbl-0009], [Table cogs70188-tbl-0010], [Table cogs70188-tbl-0011], [Table cogs70188-tbl-0012], [Table cogs70188-tbl-0013]

**Table C.1 cogs70188-tbl-0004:** Likelihood ratio tests for behavioral characteristics of Experiment 1 (causality)

Model comparison	$X^{2}$	*df*	*p*	*p* < .050
Full versus main	213.52	5.00	< .001	***
Main versus cue	243.39	5.00	< .001	***
Main versus characteristic	1.50	1.00	.220	
Cue	1.38	1.00	.241	
Characteristic	243.26	5.00	< .001	***

**p* < .050; ***p* < .010; ****p* < .001.

**Table C.2 cogs70188-tbl-0005:** Likelihood ratio tests for selfhood‐attribution of Experiment 1 (causality)

Model comparison	$X^{2}$	*df*	*p*	*p* < .050
Full versus main	101.38	8.00	< .001	***
Main versus cue only	1630.98	8.00	< .001	***
Main versus subscale only	163.05	1.00	< .001	***
Cue versus null	129.97	1.00	< .001	***
Susbcale versus null	1597.90	8.00	< .001	***

**p* < .050; ***p* < .010; ****p* < .001.

**Table C.3 cogs70188-tbl-0006:** Likelihood ratio tests for behavioral characteristics of Experiment 2 (equifinality)

Model comparison	$X^{2}$	*d* *f*	*p*	*p* < .050
Full versus main	156.69	5.00	< .001	***
Main versus cue	407.14	5.00	< .001	***
Main versus characteristic	315.74	1.00	< .001	***
Cue	275.03	1.00	< .001	***
Characteristic	366.44	5.00	< .001	***

**p* < .050; ***p* < .010; ****p* < .001.

**Table C.4 cogs70188-tbl-0007:** Likelihood ratio tests for selfhood‐attribution of Experiment 2 (equifinality)

Model comparison	$X^{2}$	*df*	*p*	*p* < .050
Full versus main	94.21	8.00	< .001	***
Main versus cue only	2607.62	8.00	< .001	***
Main versus subscale only	235.21	1.00	< .001	***
Cue versus null	163.90	1.00	< .001	***
Susbcale versus null	2536.32	8.00	< .001	***

**p* < .050; ***p* < .010; ****p* < .001.

**Table C.5 cogs70188-tbl-0008:** Likelihood ratio tests for behavioral characteristics of Experiment 3 (efficiency)

Model comparison	$X^{2}$	*df*	*p*	*p* < .050
Full versus main	326.20	5.00	< .001	***
Main versus cue	658.38	5.00	< .001	***
Main versus characteristic	160.02	1.00	< .001	***
Cue	127.24	1.00	< .001	***
Characteristic	625.60	5.00	< .001	***

**p* < .050; ***p* < .010; ****p* < .001.

**Table C.6 cogs70188-tbl-0009:** Likelihood ratio tests for selfhood‐attribution of Experiment 3 (efficiency)

Model comparison	$X^{2}$	*df*	*p*	*p* < .050
Full versus main	265.75	8.00	< .001	***
Main versus cue only	2465.16	8.00	< .001	***
Main versus subscale only	15.56	1.00	< .001	***
Cue versus null	11.01	1.00	< .001	***
Susbcale versus null	2460.61	8.00	< .001	***

**p* < .050; ***p* < .010; ****p* < .001.

**Table C.7 cogs70188-tbl-0010:** Likelihood ratio tests for behavioral characteristics of Experiment 4 (learning)

Model comparison	$X^{2}$	*df*	*p*	*p* < .050
Full versus main	122.90	5.00	< .001	***
Main versus cue	634.73	5.00	< .001	***
Main versus characteristic	268.43	1.00	< .001	***
Cue	216.03	1.00	< .001	***
Characteristic	582.33	5.00	< .001	***

**p* < .050; ***p* < .010; ****p* < .001.

**Table C.8 cogs70188-tbl-0011:** Likelihood ratio tests for selfhood‐attribution of Experiment 4 (learning)

Model comparison	$X^{2}$	*df*	*p*	*p* < .050
Full versus main	87.52	8.00	< .001	***
Main versus cue only	3115.01	8.00	< .001	***
Main versus subscale only	278.35	1.00	< .001	***
Cue versus null	180.96	1.00	< .001	***
Susbcale versus null	3017.62	8.00	< .001	***

**p* < .050; ***p* < .010; ****p* < .001.

**Table C.9 cogs70188-tbl-0012:** Likelihood ratio tests for behavioral characteristics of Experiment 5 (context)

Model comparison	$X^{2}$	*df*	*p*	*p* < .050
Full versus main	9.86	5.00	.079	
Main versus cue	385.20	5.00	< .001	***
Main versus characteristic	0.40	1.00	.525	
Cue	0.35	1.00	.553	
Characteristic	385.15	5.00	< .001	***

**p* < .050; ***p* < .010; ****p* < .001.

**Table C.10 cogs70188-tbl-0013:** Likelihood ratio tests for selfhood‐attribution of Experiment 5 (context)

Model comparison	$X^{2}$	*df*	*p*	*p* < .050
Full versus main	19.71	8.00	.011	*
Main versus cue only	3738.62	8.00	< .001	***
Main versus subscale only	0.74	1.00	.391	
Cue versus null	0.44	1.00	.509	
Susbcale versus null	3738.32	8.00	< .001	***

**p* < .050; ***p* < .010; ****p* < .001.
